# Recent trends in microbial production of alkanes

**DOI:** 10.1007/s11274-025-04536-y

**Published:** 2025-09-02

**Authors:** Noura Sh. A. Hagaggi, Usama M. Abdul-Raouf

**Affiliations:** Botany Department, Faculty of Science, Aswan University, Aswan, 81528 Egypt

**Keywords:** Alkane, Microbial production, Biofuel, Sustainable, Development

## Abstract

Alkanes are saturated hydrocarbons characterized by their stable chemical properties and multiple uses in the energy sector. Although alkanes are found in nature in various sources like natural gas and petroleum, microbial alkane production has become a promising way to develop sustainable biofuels. Alkanes are produced by microorganisms such as bacteria, fungi, yeast, and algae via particular metabolic pathways. Although progress has been achieved, challenges remain, including low conversion efficiencies and excessive costs of biofuel production compared to conventional fossil fuels, along with the need to enhance microbial resistance to harsh production conditions. By understanding current progress and future challenges, researchers and bioengineers can develop modern technologies and methods to improve the efficiency and effectiveness of microbial alkane production. This review provides a detailed look at the recent developments in microbial biosynthesis of alkanes, highlighting the key metabolic pathways and genetic techniques used to scale up production. It also discusses the challenges and obstacles facing the production of microbial alkanes. The review emphasizes the role of microbial alkane production in promoting a sustainable energy future. This review may enhance awareness of the importance of developing renewable biofuel sources, which supports global efforts to combat climate change and achieve sustainable development.

**Supplementary Information:**

The online version contains supplementary material available at 10.1007/s11274-025-04536-y.

## Introduction

Alkanes, which are saturated hydrocarbon molecules composed of hydrogen and carbon atoms, are vital feedstocks in the petrochemical industry and fundamental ingredients in fuels such as gasoline, diesel, and jet fuel. They can be straight-chain, branched-chain, or cyclic molecules and generally have the chemical formula (CₙH₂ₙ₊₂). Hexadecane (C₁₆H₃₄), octane (C₈H₁₈), and methane (CH₄) are typical examples. Alkanes are promising candidates for sustainable, renewable, and engine-compatible biofuels (Saini et al. 2023). They have a unique advantage over conventional fossil fuels in the shift to greener energy systems due to their drop-in compatibility, high energy density, cleaner emissions, and potential for carbon neutrality (Xiong et al. 2024).

Alkanes act as building blocks for chemicals. For example, ethane is used to produce ethylene, a key component of polyethylene and plastics. Heavier alkanes can be converted into alkenes and aromatics through current cracking and steam cracking processes, which are used in the production of rubber, fibers, and solvents. Additionally, alkanes are crucial in manufacturing plastics such as polyethylene and polypropylene, which are utilized in consumer products and commercial applications (Dong 2021). Agricultural residues, glucose, glycerol, and carbon dioxide are often inexpensive feedstocks for microbial alkane production (Lehtinen et al. 2018). Through engineered metabolic pathways, bacteria, yeasts, and algae can convert these feedstocks into alkanes. Advances in systems biology, metabolic engineering, and synthetic biology have significantly improved the yield, scalability, and efficiency of microbial alkane synthesis (Sharma et al. 2024).

Compared to traditional methods, microbial production of alkanes provides several strong advantages, both environmentally and economically (Cao et al. 2024). It promotes sustainability by using renewable feedstocks like agricultural waste, plant biomass, glycerol, and even carbon dioxide, which helps reduce dependence on fossil fuels. Unlike conventional petrochemical processes, microbial alkane biosynthesis takes place under mild conditions, at ambient temperatures and pressures, leading to lower energy consumption and cost savings (Hayashi and Arai 2022). Additionally, microbial systems can be genetically engineered for scalability and fine-tuning, allowing precise control over the chain length and amount of alkanes produced to meet different industrial needs (Onyeaka and Ekwebelem 2023). One major environmental benefit of microbial alkane production is its ability to significantly cut greenhouse gas emissions. Extracting and burning fossil fuels release large quantities of CO₂ and other pollutants, which heavily contribute to climate change. In contrast, engineered microorganisms that can fix CO₂ offer carbon-neutral or even carbon-negative routes for alkane synthesis, presenting a promising alternative to traditional fuels (Geng et al. 2023; Kurt et al. 2023; Tiwari et al. 2024). Moreover, bioproduction in closed, controlled bioreactor systems removes risks associated with oil spills, water pollution, and habitat loss, which are common issues with fossil fuel extraction (Lehtinen et al. 2018). By increasing the use of renewable resources and reducing environmental impact, microbial alkane production supports the goals of a circular bioeconomy and helps ensure long-term energy security and ecological health (He and Liu 2023).

From an economic standpoint, the development of microbial alkane production methods is strongly motivated by the goal of attaining market stability and energy security (Jeon et al. 2024). Energy market stability and national energy security can be improved by lowering reliance on oil supplies from geopolitically unstable regions. Additionally, the development of these technologies may improve the local economies by opening up new markets for farmers by using agricultural wastes as raw materials for production (Wang and Zhu 2018). Furthermore, investing in microbial alkane production technology can give businesses and nations a competitive edge in the expanding green economy by putting them at the forefront of biofuel innovation (Khan et al. 2021).

**Fig. 1:**
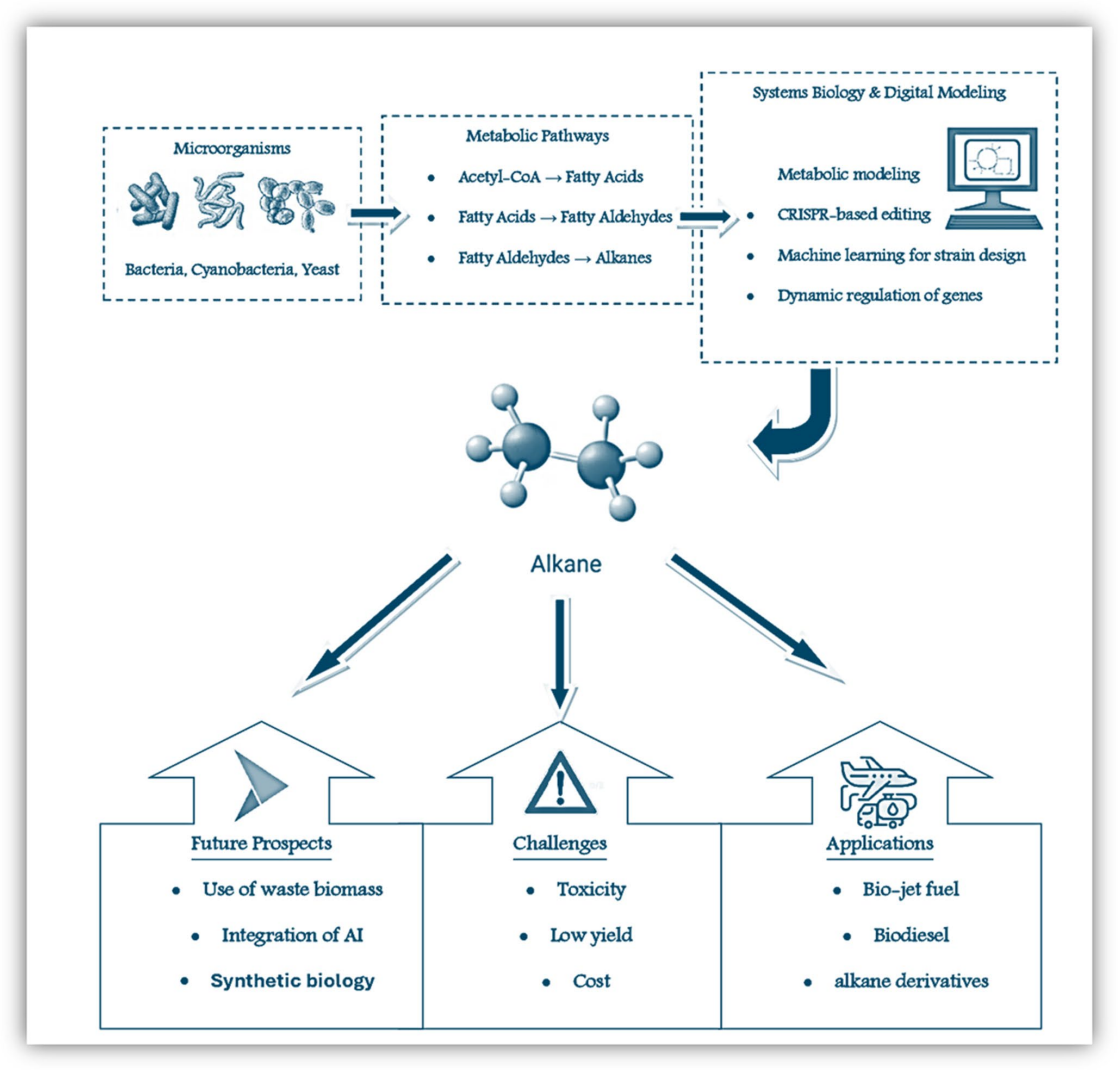
The overall microbial production of alkanes

Overall, the environmental and commercial issues related to the use of conventional fossil fuels can be greatly mitigated by the development of microbial alkane production technology (Okoro et al. 2022). Microbial production methods can significantly reduce the negative environmental effects of energy use while promoting stability and economic progress by offering a sustainable and renewable energy source (Azimov et al. 2021). These two advantages highlight how crucial it is to continue research and development in this crucial area. Therefore, this review aims to guide the recent developments in the microbial production of alkanes. The overall microbial production of alkanes was illustrated in Fig. 1.

## Microbial synthesis of alkanes

**Fig. 2:**
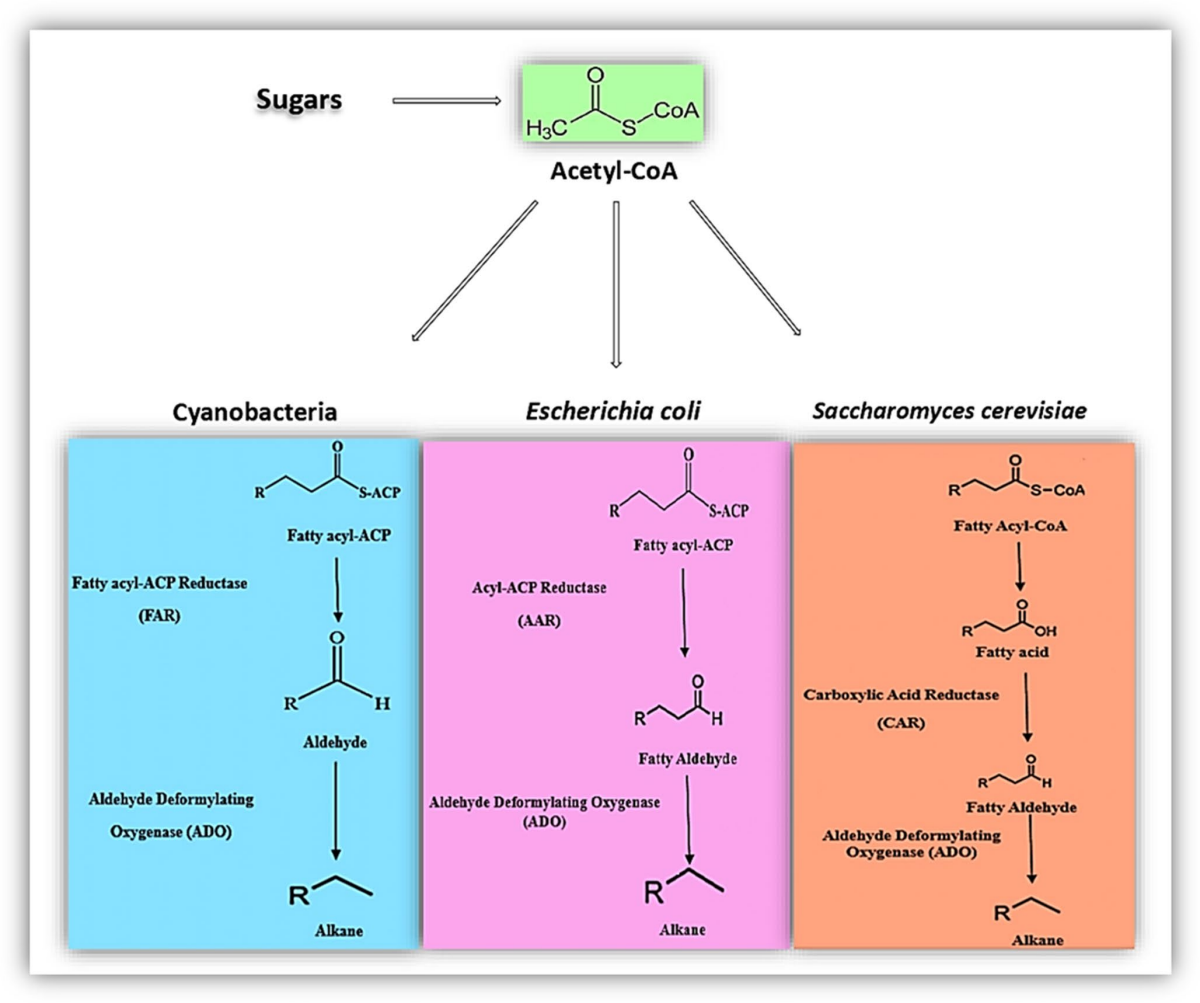
Schemes of microbial alkanes biosynthesis pathways: Cyanobacteria (Li et al. 2012), *Escherichia coli* (Song et al. 2016), and *Saccharomyces cerevisiae* (Kang et al. 2017)

Specialized enzymatic pathways are used in the microbial production of alkanes (Fig. 2), which are essential building blocks of industrial and biofuels (Zhang et al. 2011). One of the most well-established microbial alkane production routes is the fatty acid-derived pathway, which involves two key enzymes, Acyl-ACP Reductase and Aldehyde Decarbonylase, that are essential. Acyl-ACP reductase (also known as acyl-acyl carrier protein reductase) is the enzyme responsible for the first important step in the conversion of primary fatty acids to alkanes (Schirmer et al. 2010). This enzyme reduces acyl-ACPs (acyl carrier protein-bound fatty acid intermediates) to form fatty aldehydes. Acyl-ACP molecules are key intermediates in fatty acid metabolism, transferring electrons to their reduction by acyl-ACP reductase, usually from NADPH to an acyl-ACP substrate, resulting in fatty aldehyde formation. This step is important because it gives fatty aldehydes after alkane biosynthesis (Brown et al. 2018). It is the first direct requirement for the term. Once a fatty aldehyde is formed, it is converted to base by aldehyde decarbonylase (often abbreviated as ADC). Aldehyde decarbonylase is a decarbonylation reaction, where the carbonyl group of an aldehyde is removed as carbon monoxide (CO) or carbon dioxide (CO2), depending on the particular enzyme and organism (Choi and Lee 2013). This reaction efficiently removes one carbon atom from an aldehyde, leading to the formation of an alkane. This enzymatic step is essential for the final synthesis of alkanes, linking fatty acid metabolism to hydrocarbon synthesis (Song et al. 2016).

Polyketide Synthase (PKS)-Like Pathways involves Type I polyketide synthase (PKS)-like enzymes, which can form hydrocarbon chains de novo from basic substrates such as malonyl-CoA. These pathways, found in *Nostoc punctiforme* and certain actinobacteria, operate independently of fatty acid precursors, giving them a feedstock flexibility advantage. Ols is one such enzyme that uses iterative chain elongation and decarboxylation to produce long-chain alkanes and alkenes. Efforts in synthetic biology to utilize modular PKS systems for customized alkane production have been driven by this biosynthetic mechanism. Heterologous expression of these PKS-like gene clusters in Streptomyces and *E. coli* has yielded encouraging titers of medium-chain hydrocarbons (Risdian et al. 2019).

In another pathway, free fatty acids are directly converted into terminal alkenes and alkanes through oxidative decarboxylation by cytochrome P450 fatty acid decarboxylases such as OleTJE. This process requires a cofactor like hydrogen peroxide or electron transfer proteins. This pathway is used by *E. coli* and *Yarrowia lipolytica*, to produce medium and long-chain hydrocarbons (Liu et al. 2014).

### Genetically engineered microorganisms for alkane synthesis

Genetically engineered microorganisms (GEMs) are crucial in alkane production by applying synthetic strategies and optimizing synthesis methods (Table 1). GEMs are commonly used to boost alkane production by introducing, optimizing, or overexpressing key biosynthetic genes, rerouting metabolic flux toward hydrocarbon pathways, and enhancing enzyme efficiency. Often, genes encoding acyl-ACP reductase (AAR) and aldehyde decarbonylase (ADO) from alkane-producing cyanobacteria are expressed in non-native hosts such as *Escherichia coli* and *Saccharomyces cerevisiae*, allowing them to convert fatty acid intermediates into alkanes. For example, *E. coli* engineered with *Synechococcus elongatus* AAR and ADO genes can produce C₁₃–C₁₇ alkanes from glucose, while yeast strains modified to express plant-derived fatty acyl-CoA reductases and cyanobacterial ADO are tailored to produce long-chain alkanes suitable for drop-in biofuels. Additional methods include knocking out competing pathways (e.g., β-oxidation) to increase precursor resources, engineering fatty acid biosynthesis for specific chain lengths, and improving electron transfer systems to enhance ADO activity. Synthetic biology techniques, such as modular pathway assembly and promoter engineering, have further increased yields; for example, *E. coli* strains optimized with strong promoters and balanced cofactors have achieved multiple-fold increases in alkane output. Recently, oleaginous yeasts like *Yarrowia lipolytica* have been engineered to amass large lipid stores and express alkane biosynthesis genes, combining high substrate availability with strong industrial performance, making GEM-based systems a leading platform for sustainable alkane production (Warui et al. 2011; Akhtar et al. 2013; Wang et al. 2013; Kang and Nielsen 2017; Li et al. 2020; Cho et al. 2022).

**Table 1:** Alkanes biosynthesis by genetically engineered microorganisms

Host microorganism	Genetic engineering strategy	Alkane Titer	Reference
*Escherichia coli*	Heterologous expression of cyanobacterial AAR and ADO genes from *Synechocystis*; enhanced fatty-acid flux	~ 300 mg/L (C13–C17)	Warui et al. 2011
*Escherichia coli*	Engineering a synthetic cascade combining CAR (from *Mycobacterium*), FAR, and ADO	~ 2–5 mg/L	Akhtar et al. 2013
*Yarrowia lipolytica*	Expression of the codon-optimized fatty acid photodecarboxylase (*Cv*FAP)	15.3 mg/L	Li et al. 2020
*Synechococcus* sp. PCC 7002	Overexpression of native AAR and ADO pathway via heterologous gene expression	Up to ~ 5% of cell dry weight	Wang et al. 2013
*Saccharomyces cerevisiae*	Expression of insect CYP4G1 and CPR to convert fatty aldehydes to alkanes	~ 0.2 mg/L/OD₆₀₀ (C23–C27)	Kang and Nielsen 2017

*AAR* Acyl-ACP Reductase, *ADO* Aldehyde Deformylating Oxygenase, *CAR* Carboxylic Acid Reductase, FAR Fatty Acyl Reductase, *CYP4G1* Cytochrome P450 4G1, and *CPR* Cytochrome P450 Reductase

Clustered Regularly Interspaced Short Palindromic Repeats (CRISPR) and other advanced genetic engineering tools have become essential for improving microbial alkane biosynthesis by enabling precise, efficient, and multiplex genome modifications that optimize metabolic pathways. CRISPR–Cas systems allow targeted insertion, deletion, or replacement of genes involved in fatty acid synthesis, precursor supply, and alkane-converting enzymes such as acyl-ACP reductase (AAR) and aldehyde decarbonylase (ADO). This precision facilitates the elimination of competing pathways, such as β-oxidation or unwanted lipid storage, thereby channeling more carbon flux toward alkane production. Additionally, CRISPR enables fine-tuning of promoter strength, ribosome binding sites, and regulatory elements to balance enzyme expression and prevent metabolic bottlenecks. Beyond CRISPR, tools like λ-Red recombineering, TALENs, and multiplex automated genome engineering (MAGE) provide complementary strategies for large-scale pathway optimization and rapid strain diversification. These approaches can also be used to introduce heterologous genes from cyanobacteria, algae, or plants into robust industrial hosts like *Escherichia coli*, *Saccharomyces cerevisiae*, or *Yarrowia lipolytica*. By integrating CRISPR with high-throughput screening and omics-guided metabolic modeling, researchers can iteratively design and test strains with improved enzyme kinetics, cofactor balance, and precursor supply. Such precise and accelerated strain engineering has significantly boosted alkane titers and process efficiency, making CRISPR-based genome editing a cornerstone in developing commercially viable microbial biofuel platforms (Ansori et al. 2023; Garg et al. 2023; Yook and Alper 2025).

To optimize alkane yields, additional improvements include the incorporation of synthetic transcriptional regulators and feedback-resistant enzymes. For industrial biofuel applications, GEMs can attain increased productivity, less byproduct formation, and enhanced scalability by combining these techniques (Hayashi and Arai 2022).

## Metabolic pathway improvement

The advancement of microbial metabolic pathways for alkane production is an expanding research area focused on creating sustainable and environmentally friendly alternatives to fossil fuels (Kang and Nielsen 2017). Microorganisms such as *Escherichia coli*, *Saccharomyces cerevisiae*, *Yarrowia lipolytica*, and cyanobacteria have been genetically modified to produce alkanes by introducing or enhancing specific biosynthetic pathways (Sharma and Yazdani 2021). A key strategy involves incorporating essential foreign enzymes, such as acyl-ACP reductase (AAR), which transforms fatty acyl-ACP into fatty aldehydes, and aldehyde-deformylating oxygenase (ADO), which then converts these aldehydes into alkanes, including pentadecane and heptadecane. However, the low activity and oxygen sensitivity of some of these enzymes, especially ADO, require further engineering to boost efficiency (Wang and Lu 2013). To systematically outline the current efforts in this field, the key strategies employed to enhance microbial alkane production were summarized (Table 2).

**Table 2:** Strategies for enhancing microbial alkane production

Strategy	Description	Tools/Approaches	Reference
Pathway enzyme engineering	Improve the activity/stability of AAR and ADO	Directed evolution, rational design, fusion proteins	Xu et al. 2022
Precursor supply enhancement	Boost fatty acid intermediates	Overexpress ACC, FAS; enhance fatty acid synthesis	Davis et al. 2000
Competing pathway suppression	Redirect flux toward alkanes	Knockout of β-oxidation genes	Ku et al. 2020
Dynamic regulation	Fine-tune gene expression	Inducible promoters, riboswitches, and feedback inhibition	Tan and Prather 2017
Cofactor optimization	Ensure reducing power and oxygen availability	NADPH enhancement (PPP pathway), oxygen control	Chemler et al. 2010
Protein scaffolding	Improve metabolic flux between steps	Enzyme scaffolds, substrate channeling	Andre et al. 2013
Membrane and efflux adaptations	Increase tolerance to alkanes	Membrane remodeling, efflux pumps	Chen et al. 2013
Host organism selection	Use robust or oleaginous microbes	e.g., *Yarrowia lipolytica*	Fukuda 2023
Advanced synthetic biology	Precision control and host optimization	CRISPR/Cas9, modeling, machine learning, synthetic circuits	Teng et al. 2024
Integrated platform engineering	Combine all strategies for industrial-scale production	Systems biology, omics, and synthetic biology	Joshi and Mishra 2022

*AAR* Acyl-ACP Reductase, *ADO* Aldehyde-Deformylating Oxygenase, *ACP* Acyl Carrier Protein, *ACC* Acetyl-CoA Carboxylase, *FAS* Fatty Acid Synthase, *NADPH* Nicotinamide Adenine Dinucleotide Phosphate, *CRISPR* Clustered Regularly Interspaced Short Palindromic Repeats

To enhance alkane yield, it is crucial to optimize the supply of metabolic precursors. This often involves increasing the flux through the fatty acid synthesis pathway by overexpressing enzymes such as acetyl-CoA carboxylase (ACC) and fatty acid synthase (FAS), which boost the intracellular concentration of fatty acid intermediates (Davis et al. 2000). At the same time, competing metabolic pathways that degrade fatty acids, such as β-oxidation, are commonly downregulated or knocked out to redirect carbon flux toward alkane biosynthesis (Ku et al. 2020). Dynamic regulation tools are also employed to fine-tune pathway expression. For instance, inducible promoters allow temporal control of gene expression, enabling the separation of cell growth and production phases. Synthetic riboswitches and feedback inhibition mechanisms help balance the expression of key enzymes and prevent the accumulation of toxic intermediates, improving both cellular health and production efficiency (Tan and Prather 2017).

Cofactor availability, particularly NADPH and oxygen, plays a significant role in alkane biosynthesis (Chemler et al. 2010). Since AAR and ADO require reducing equivalents and, in the case of ADO, molecular oxygen, metabolic engineering efforts often focus on enhancing NADPH-generating pathways like the pentose phosphate pathway or adjusting culture conditions to ensure optimal oxygen levels. Additionally, protein engineering techniques such as directed evolution and rational design are applied to improve enzyme stability, activity, and substrate specificity (Xu et al. 2022). Creating fusion proteins or enzyme scaffolds can also increase the efficiency of substrate channeling between consecutive reactions, thus enhancing overall flux through the alkane biosynthetic pathway (Andre et al. 2013).

Microbial tolerance to alkanes is another key factor that impacts production. Since alkanes are hydrophobic and can disrupt cell membranes, several strategies have been employed to overcome this limitation and enhance microbial tolerance (Chen et al. 2013). Membrane engineering is a common approach, where modifications in fatty acid composition or the introduction of genes involved in lipid remodeling help maintain membrane stability in the presence of hydrophobic products. For example, increasing the proportion of saturated fatty acids or introducing cyclopropane fatty acids can reduce membrane fluidity and leakage. Efflux pump engineering is another widely used tactic; heterologous or native solvent-efflux systems such as AcrAB-TolC in *Escherichia coli* or SrpABC in *Pseudomonas putida* can actively transport alkanes out of the cell, thereby lowering intracellular concentrations and mitigating toxicity (Pabbathi et al. 2025). Adaptive laboratory evolution (ALE) has also been effective by gradually exposing microbial cultures to increasing alkane concentrations over many generations; more tolerant strains can be selected, often with mutations in stress response regulators, membrane biosynthesis genes, or efflux transporters. In addition, global transcription machinery engineering (gTME) and regulatory network rewiring have been employed to reprogram stress response pathways and upregulate genes involved in membrane repair, chaperone activity, and detoxification. Another promising method is two-phase partitioning bioprocesses, where alkanes are continuously extracted into a non-toxic second phase (e.g., biocompatible solvents or immiscible oils), preventing excessive intracellular accumulation (Wang et al. 2023). Together, these strategies reduce product-associated stress, improve cell viability during production, and enable higher titers and yields in industrial alkane biosynthesis systems. Some research also targets the use of naturally robust or oleaginous microorganisms, such as *Yarrowia lipolytica*, which are more tolerant to hydrocarbons and have an inherent ability to accumulate lipids, making them ideal hosts for alkane production (Fukuda 2023).

Recent advancements, including CRISPR/Cas9 genome editing, computational modeling, and machine learning, have further accelerated the rational design and optimization of microbial alkane producers. These tools allow for precise, large-scale genome modifications and predictive optimization of metabolic networks (Teng et al. 2024). When combined with synthetic biology approaches, they open the door to developing industrially viable microbial platforms capable of producing high yields of alkanes from renewable feedstocks, such as glucose, glycerol, and even CO₂ through photosynthetic organisms (Decker et al. 2023). The integration of pathway engineering, dynamic control, and host optimization forms the basis for advancing microbial alkane production toward commercial-scale biofuel applications (Joshi and Mishra 2022).

## Systems biology and digital modeling

Systems biology and digital modeling are crucial for understanding and improving alkane production by microorganisms (Casey et al. 2024). Since microbial alkane biosynthesis involves complex, interconnected metabolic pathways, systems biology offers a comprehensive approach to examining these interactions at multiple levels-genomic, transcriptomic, proteomic, and metabolomic (Qiao et al. 2025). By integrating data from these layers, researchers can develop detailed models of cellular behavior, helping them identify key regulatory points, metabolic bottlenecks, and ideal intervention targets to increase alkane output (Cao et al. 2020).

Digital modeling, particularly through genome-scale metabolic models, allows simulation of the entire metabolic network of an organism under different genetic and environmental conditions (Moyer et al. 2025). These models predict the effects of gene knockouts, overexpression, or pathway insertions on cellular metabolism and alkane output. Flux balance analysis (FBA), a widely used computational technique, enables the calculation of intracellular metabolic fluxes based on stoichiometric constraints, facilitating the identification of optimal metabolic states for alkane biosynthesis (Tarzi et al. 2024).

Runguphan and Keasling (2014) employed an integrated multi-omics approach, combining transcriptomic and proteomic analyses, to systematically redesign the fatty acid pathway in *Saccharomyces cerevisiae*. This strategy successfully redirected fatty acyl-CoA flux toward alkane biosynthesis, overcoming competing metabolic routes. Their work marked the first successful demonstration of alkane production in *S. cerevisiae*, laying the groundwork for future improvement of yeast-based biofuel production. Fatma et al. (2018) used genome-scale metabolic modeling (GEM) in *Escherichia coli* to improve the production of fatty alcohols and alkanes. The model helped identify key metabolic bottlenecks, such as limited NADPH supply and inefficiencies in the β-oxidation pathway, which were then addressed through genetic and process engineering. This model-driven approach resulted in impressive titers, reaching up to 2.54 g/L of alkanes in fed-batch fermentation, demonstrating the effectiveness of computational tools in designing efficient biofuel pathways. Coupling GEMs and omics approaches further enhances predictive power and accuracy (Bernstein et al. 2023).

Alongside static modeling, dynamic modeling tools like ordinary differential equations (ODEs) are employed to depict changes over time in metabolite levels and enzyme activities (Yang et al. 2019). These models are especially valuable for understanding processes that depend on time, such as substrate uptake, intermediate buildup, and product synthesis (Liu et al. 2018). Including regulatory features like transcriptional control, feedback inhibition, and post-translational modifications in these models offers a more accurate depiction of cellular behavior and facilitates the simulation of dynamic regulation methods used in synthetic biology (Zhou and Yan 2024).

Particularly for simulating diverse populations or microbial consortia designed for the production of alkanes, agent-based models and hybrid models that incorporate both stochastic and deterministic components are also becoming more and more popular (Hunter et al. 2020). Researchers can now model population dynamics, individual cell behavior, and interactions between engineered strains with complementary metabolic roles thanks to these sophisticated tools (Liao et al. 2019). Computational modeling and artificial intelligence (AI) have become powerful tools in the design of efficient microbial strains for alkane production by enabling precise, data-driven optimization of metabolic pathways before costly laboratory experiments (Dasgupta and De 2023). They allow researchers to simulate cellular metabolism under various genetic and environmental conditions, identify bottlenecks in precursor supply, and predict the effects of targeted gene modifications on alkane yield (Mao et al. 2024).

By directing hypothesis-driven research, systems biology and digital modeling not only make it easier to rationally design microbial strains for increased alkane production, but they also lessen the experimental burden (Gargalo et al. 2024). A foundational framework for scaling up microbial alkane production in industrial bioprocesses is provided by this integrated approach, which also minimizes expensive trial-and-error experiments and speeds up strain development cycles (Isoko et al. 2024). The beneficial relationship between systems biology and digital modeling will be essential for developing microbial biofuel technology as data generation speeds up and computational tools become more advanced (Gowen and Fong 2011).

## Advanced industrial applications

Alkane production by microbes has drawn more and more interest as a sustainable substitute for fuels derived from fossil fuels, especially in light of climate change and energy security (Holechek et al. 2022). The substantial advancements in this field, from lab-scale viability to pilot and industrial-scale implementation, are illustrated by a number of case studies and industrial applications (Piccinno et al. 2016). For instance, using engineered *Saccharomyces cerevisiae*, the biotechnology company Amyris first concentrated on the microbial synthesis of farnesene, a sesquiterpene with characteristics akin to diesel (Rautela et al. 2024). The commercial viability of using engineered microbes to produce hydrocarbon-like compounds was established by the production of farnesene, although it is not an alkane. Building on this basis, research has shifted to modifying bacterial pathways, including those of cyanobacteria and *Escherichia coli*, to directly produce straight-chain alkanes from sugars or CO₂ (Srivastava et al. 2025; Yao et al. 2020).

A further interesting case involved the engineering of *E. coli* strains to produce fatty acid-derived alkanes by a pathway involving acyl-ACP thioesterases, fatty aldehyde reductases, and aldehyde-deformylating oxygenases (ADO) by the company LS9 Inc., which was later acquired by Renewable Energy Group (Coursolle et al. 2015). The microbial conversion of sugars derived from biomass into drop-in fuels, such as diesel, was made possible by this process (Peoples et al. 2022). Alkanes could be continuously produced in an industrial setting thanks to LS9’s optimization of fermentation and metabolic pathways (Westfall and Gardner 2011). In order to improve yield and scalability, the process also included phase separation techniques for downstream recovery (Cuellar and Straathof 2020). Even though problems like ADO’s oxygen sensitivity and enzyme inefficiency still exist, LS9’s work laid the groundwork for the development of process engineering strategies to lessen these problems (Azimov et al. 2021).

Moreover, large-scale efforts are underway through collaborative projects between academia and industry (Tanaka and Lopez 2024). The U.S. Department of Energy’s Joint BioEnergy Institute (JBEI) has engineered strains of *E. coli* and *Yarrowia lipolytica* capable of producing medium-chain alkanes at titers nearing industrial relevance (Rutter et al. 2015). JBEI combines computational modeling, machine learning, and automated strain construction platforms to develop more robust microbial cell factories (Wehrs et al. 2019). These systems utilize advanced bioprocess controls, real-time metabolite monitoring, and innovations in downstream purification to support a transition toward commercial readiness (Gargalo et al. 2020).

Overall, these studies showcase a growing ecosystem of innovation in microbial alkane production. While no company has yet fully commercialized microbial alkane fuels at a large scale due to economic and regulatory hurdles, advancements in strain engineering, pathway optimization, and bioprocess design indicate strong potential for future adoption (Okoro et al. 2022). As global investment in bio-based fuels increases and environmental regulations tighten, these technologies are well-positioned to become key parts of the next-generation bioeconomy (Kamalesh et al. 2024).

## Challenges and limitations

Microbial alkane production at an industrial scale faces several technical, biological, and economic challenges that currently limit its commercial viability (Geng et al. 2023). A major bottleneck lies in low production titers, yields, and productivities, as native microbial pathways for alkane biosynthesis, particularly those involving aldehyde decarbonylase (ADO), are slow and energetically demanding, often resulting in insufficient conversion rates for large-scale applications (Fatma et al. 2018; Liu et al. 2021). Enzyme inefficiency, poor substrate specificity, and limited tolerance to industrial fermentation conditions further constrain pathway performance (Hayashi and Arai 2022).

Another major challenge is product toxicity. Alkanes, especially in longer-chain forms, are hydrophobic compounds that can accumulate within microbial cell membranes, leading to membrane disruption and cellular stress. This toxicity reduces microbial growth and productivity over time (Libisch 2024). While some strategies, such as exporting alkanes into a separate phase or engineering more tolerant microbial membranes, have shown promise, they are not universally effective across different production systems or scales (Zhiwei et al. 2017). Moreover, the extraction of alkanes from fermentation broth poses downstream processing difficulties, as alkanes are typically insoluble in water and require energy-intensive separation techniques, such as solvent extraction or distillation, which further increase production costs (Janković et al. 2024).

Feedstock cost and sustainability also pose important limitations. Many microbial alkane production systems rely on sugars derived from starch or lignocellulose, which can compete with food supplies or require complex pretreatment steps (Mansy et al. 2025). While photoautotrophic organisms like cyanobacteria offer an alternative by utilizing carbon dioxide and sunlight, they suffer from low photosynthetic efficiencies and slow growth rates, which constrain large-scale deployment. Engineering faster-growing photosynthetic microbes or co-culturing systems remains a complex and unresolved issue in the field (Agarwal et al. 2022).

Scale-up challenges remain a significant barrier. Microbial systems that perform well in laboratory-scale bioreactors often encounter unforeseen obstacles at industrial scales, such as changes in oxygen transfer rates, mixing efficiency, and shear stress (Crater and Lievense 2018). These factors can dramatically impact microbial health and productivity. Bioreactor design must be carefully adapted to the specific needs of alkane-producing microbes, requiring considerable investment in pilot testing and process engineering (de Mello et al. 2024).

Lastly, economic and regulatory hurdles cannot be overlooked. The current market price of petroleum-derived fuels is relatively low, making it difficult for bio-based alkanes to compete without substantial subsidies or carbon pricing mechanisms (van den Bergh et al. 2023). Additionally, regulatory approval for new microbial strains, especially genetically modified organisms, can be a lengthy and uncertain process, depending on the region. These policy and market dynamics create uncertainty for investors and companies aiming to commercialize microbial alkane production (Shams et al. 2024).

### Visions and future prospects

Alkanes produced by microbes have great potential as a renewable and sustainable substitute for hydrocarbons derived from fossil fuels. In the fight against climate change and resource depletion, microbes like cyanobacteria, yeast, and genetically modified bacteria present a novel platform for biologically producing alkanes, which are essential components of gasoline, diesel, and jet fuels (Mund et al. 2022; Hagaggi and Rady 2024). Scientists believe that microbial alkane production will be a key component of the next-generation biofuel industries as synthetic biology, metabolic engineering, and systems biology continue to advance (Han et al. 2023). These technologies increase the efficiency and commercial viability of biological systems by enabling precise control over metabolic pathways, optimizing carbon flux toward alkane biosynthesis, and improving microbial tolerance to toxic end products (Parveen and Yazdani 2022). Looking ahead, microbial alkane production holds strong promise for sustainable energy systems by reducing greenhouse gas emissions, utilizing renewable feedstocks such as CO₂ or agricultural waste, and minimizing reliance on non-renewable petroleum resources. On an industrial scale, further breakthroughs in strain engineering, process optimization, and cost-effective downstream recovery are expected to enable large-scale commercialization, positioning microbe-derived alkanes as a viable competitor to fossil fuels in transportation, aviation, and other heavy-duty energy sectors (Yılbaşı 2025).

In the future, it is anticipated that the combination of computational modeling, machine learning, and artificial intelligence will be crucial in improving the layout and functionality of microbial cell factories (Mohseni and Ghorbani 2024). Building modular, programmable microbial strains that can produce specific alkane profiles for various fuel grades and industrial applications is probably the main focus of future research (He et al. 2023). Simultaneously, it is expected that the development of reliable, large-scale bioreactors and inexpensive feedstock conversion systems, such as the use of lignocellulosic biomass or industrial CO₂ emissions, will greatly reduce production costs and their negative effects on the environment. Carbon-neutral feedstocks and engineered microbes could lead to closed-loop systems that help sequester carbon and promote energy sustainability (Chowdhury et al. 2025).

Additionally, current bottlenecks related to metabolic burden and pathway inhibition may be overcome by incorporating dynamic regulatory systems and synthetic consortia, in which various microbial species collaborate (Ibrahim et al. 2021). More robust and adaptable production platforms that can adjust to shifting industrial and environmental conditions may be made possible by these strategies (Rame et al. 2024). In the future, there may also be decentralized, on-site alkane production facilities that use engineered microbes to turn local biomass into fuel, particularly in rural or isolated areas. This would improve energy security and lower transportation costs (Trisrivirat et al. 2020).

Ultimately, microbial alkane production seems to have a promising future, despite ongoing obstacles like public acceptance, regulatory barriers, and economic scalability (Lim et al. 2023). To turn these scientific ideas into workable, scalable solutions, microbiologists, engineers, chemists, and environmental scientists will need to continue their interdisciplinary collaboration. Microbial alkane production has the potential to transform the energy industry and make a substantial contribution to the achievement of global climate goals if it is successful (Manikandan et al. 2022).

## Conclusion

The microbial production of alkanes is a promising field in biotechnology, synthetic biology, and sustainable energy development. Advances in metabolic pathway engineering, systems biology, and dynamic gene regulation have improved alkane yield, selectivity, and process efficiency across various microbial hosts. The integration of computational design and high-throughput screening technologies has accelerated the development of microbial cell factories for alkane biosynthesis. Despite challenges like metabolic burden, product toxicity, low productivity, and scalability, ongoing research is paving the way for future breakthroughs.

## Supplementary Information

Below is the link to the electronic supplementary material.

Supplementary Material 1

## Data Availability

No datasets were generated or analysed during the current study.
