# Supplementary figures and images for: Recent trends in microbial production of alkanes
Source: World J Microbiol Biotechnol. 2025 Sep 2;41(9):320. doi: 10.1007/s11274-025-04536-y (PMC12405031; doi:10.1007/s11274-025-04536-y)

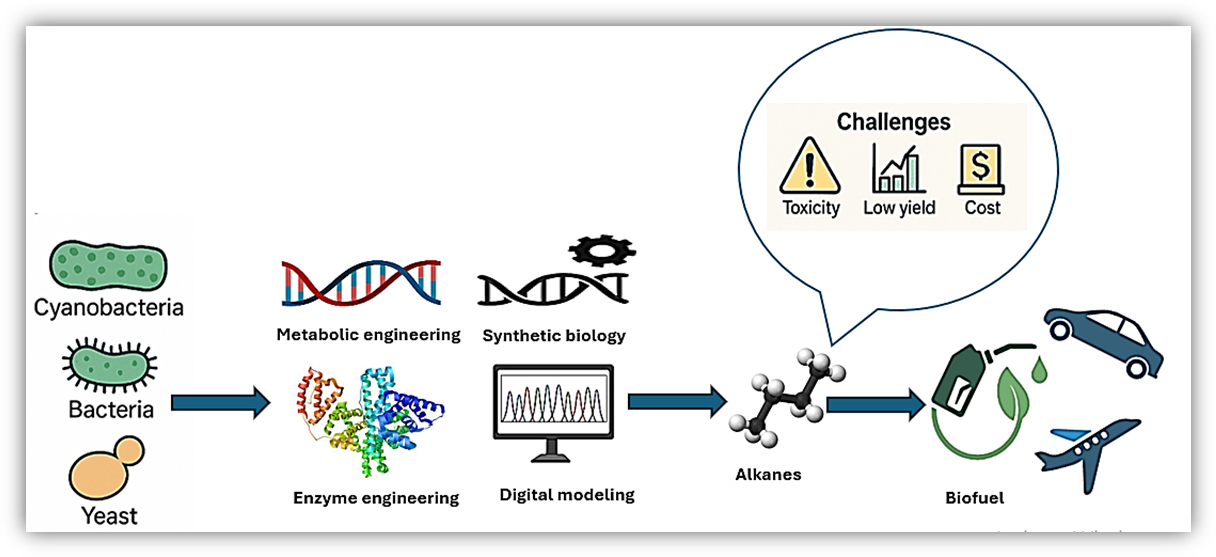

Supplement: Supplementary file 1 — Supplementary Material 1 [file 11274_2025_4536_MOESM1_ESM.doc]
